# The tumor marker Fascin is induced by the Epstein-Barr virus-encoded oncoprotein LMP1 via NF-κB in lymphocytes and contributes to their invasive migration

**DOI:** 10.1186/s12964-014-0046-x

**Published:** 2014-07-11

**Authors:** Caroline F Mohr, Martina Kalmer, Christine Gross, Melanie C Mann, Kai R Sterz, Arnd Kieser, Bernhard Fleckenstein, Andrea K Kress

**Affiliations:** 1 Institute of Clinical and Molecular Virology, Friedrich-Alexander-Universität Erlangen-Nürnberg, Erlangen, Germany; 2 Research Unit Gene Vectors, Helmholtz Zentrum München - German Research Center for Environmental Health, München, Germany; 3 German Center for Infection Research (DZIF), Partner Site Munich, Marchioninistr. 25, Munich, D-81377, Germany

**Keywords:** NF-κB, Fascin, Epstein-Barr virus, LMP1, CTAR, Invasion, Migration

## Abstract

**Background:**

The actin-bundling protein Fascin (FSCN1) is a tumor marker that is highly expressed in numerous types of cancer including lymphomas and is important for migration and metastasis of tumor cells. Fascin has also been detected in B lymphocytes that are freshly-infected with Epstein-Barr virus (EBV), however, both the inducers and the mechanisms of Fascin upregulation are still unclear.

**Results:**

Here we show that the EBV-encoded oncoprotein latent membrane protein 1 (LMP1), a potent regulator of cellular signaling and transformation, is sufficient to induce both Fascin mRNA and protein in lymphocytes. Fascin expression is mainly regulated by LMP1 via the C-terminal activation region 2 (CTAR2). Block of canonical NF-κB signaling using a chemical inhibitor of IκB kinase β (IKKβ) or cotransfection of a dominant-negative inhibitor of IκBα (NFKBIA) reduced not only expression of p100, a classical target of the canonical NF-κB-pathway, but also LMP1-induced Fascin expression. Furthermore, chemical inhibition of IKKβ reduced both *Fascin* mRNA and protein levels in EBV-transformed lymphoblastoid cell lines, indicating that canonical NF-κB signaling is required for LMP1-mediated regulation of Fascin both in transfected and transformed lymphocytes. Beyond that, chemical inhibition of IKKβ significantly reduced invasive migration of EBV-transformed lymphoblastoid cells through extracellular matrix. Transient transfection experiments revealed that Fascin contributed to LMP1-mediated enhancement of invasive migration through extracellular matrix. While LMP1 enhanced the number of invaded cells, functional knockdown of Fascin by two different small hairpin RNAs resulted in significant reduction of invaded, non-attached cells.

**Conclusions:**

Thus, our data show that LMP1-mediated upregulation of Fascin depends on NF-κB and both NF-κB and Fascin contribute to invasive migration of LMP1-expressing lymphocytes.

## Background

The DNA virus Epstein-Barr virus (EBV), also termed Human herpesvirus 4 (HHV-4), infects both B lymphoid cells and epithelial cells. EBV infections are associated with cancer as EBV DNA is detected in nearly all cases of endemic Burkitt lymphoma (BL), nasopharyngeal carcinoma (NPC) and, frequently, in Hodgkin lymphomas (HL) [[1]]. After an initial lytic phase of EBV infection, a life-long latency period is established. According to the latency phase of EBV-associated malignancies, different latent genes are expressed [[2]]. In latency type I, which is represented by BL, only EBNA-1, EBER-and BART-RNAs are expressed, while in latency type II, which is typical for HL, NPC, gastric cancer and T-cell lymphomas, also latent membrane protein 1 (LMP1) and 2A (LMP-2A) are expressed. Additionally, type III latency, which occurs in post-transplantation lymphoproliferative disease, is also characterized by the expression of LMP1 and a variety of other latency-associated viral genes [[2]]. Lymphoblastoid cell lines (LCLs) serve as a model system for type III latency. LCLs are usually derived from Epstein-Barr virus (EBV) infection of resting human B lymphocytes *in vitro*, resulting in continuous cell proliferation and transformation.

Among the virus-encoded genes, LMP1 plays a critical role in EBV-induced cellular transformation [[1]–[4]]. The LMP1 oncoprotein, encoded by the *BNLF-1* gene of EBV, constitutes a transmembrane protein composed of 386 amino acids (aa) that contributes to the development of EBV-associated tumors. Functionally, LMP1 mimics the human CD40 receptor, a costimulatory receptor of the tumor necrosis factor (TNF) receptor superfamily [[5]]. In contrast to the ligand-dependent CD40, LMP1 drives proliferation of infected B-cells independent of a ligand by spontaneous formation of LMP1 oligomers. Two carboxyterminal cytoplasmic signaling domains, the C-terminal activation regions 1 (CTAR1; aa 194–231) and 2 (CTAR2; aa 351–386), are involved in activation of signaling pathways [[6],[7]]. CTAR1 binds through a P(204)xQxT/S consensus motif to TNF receptor-associated factors (TRAFs), thereby inducing noncanonical (alternative) NF-κB signaling through NF-κB-inducing kinase (NIK) and I-κB kinase α (IKKα) [[8]–[11]]. Moreover, CTAR1 activates the p38 mitogen-activated protein kinase (MAPK), the phosphatidylinositol 3-kinase (PI3-kinase)/Akt pathway, and can contribute to activation of the c-Jun N-terminal kinase (JNK) pathway [[12]–[14]]. The signaling domain CTAR2 binds through tyrosine residue Tyr384 to TNF-receptor associated death domain (TRADD), which is required for canonical (classical) NF-κB activation and B lymphocyte transformation [[8],[15],[16]]. TRAF6 and the tumor necrosis factor-receptor-associated factor 2 (TRAF2)- and Nck-interacting kinase TNIK have critical functions in NF-κB signaling downstream of CTAR2 [[12],[17],[18]]. Additionally, CTAR2 contributes to activation of p38 MAPK [[12]] and triggers the JNK pathway [[19]].

The mechanisms by which LMP1 promotes tumorigenesis are not fully understood. In addition to LMP1-mediated alterations in cell growth and gene expression, LMP1 also increases the expression of cytoskeletal proteins and adhesion molecules [[20]], interacts with cytoskeletal components like vimentin [[21]], and causes plasma membrane ruffling and villous projections [[22]]. In EBV-transformed lymphocytes, the actin-bundling protein Fascin (FSCN-1) is overexpressed in LCLs, while it is absent in EBV-positive cell lines derived from BL [[23]]. Moreover, Fascin is a possible prognostic marker of HL independent of the presence of EBV [[24]], and it is upregulated in tissues of NPC [[25],[26]]. Fascin usually stabilizes filamentous actin and is concentrated in cellular protrusions like filopodia during cell migration [[27],[28]]. In healthy individuals, Fascin is expressed in dendritic, neuronal, mesenchymal and endothelial cells, while it is absent from epithelial cells and lymphocytes [[27],[29]]. In contrast, Fascin is upregulated in many human carcinomas including breast, lung, colon, esophagus, pancreatic, stomach, ovary, and skin cancers. Fascin is concentrated in the leading edge of cancer tissue, stabilizes invadopodia, and mediates self-seeding of cancer cells [[28],[29]]. We could previously show that silencing of Fascin decreases not only the migratory and invasive capacity of cancer cells [[28],[29]], but also the invasion rate of cells derived from Adult T-cell leukemia/lymphoma [[30]]. Recently, Fascin has received attention as a potential prognostic marker and therapeutic target for metastasis [[29],[31]].

Though there has been evidence for an association between EBV-infection and Fascin expression, both the mechanism of Fascin upregulation by EBV in lymphocytes and Fascin’s function are still unclear. In this study we show that LMP1 is sufficient to induce the tumor marker Fascin in lymphocytes depending on NF-κB signaling. We provide evidence that Fascin contributes to LMP1-mediated invasive migration.

## Results

### Fascin is differentially expressed in transformed lymphocytes

**Figure 1:**
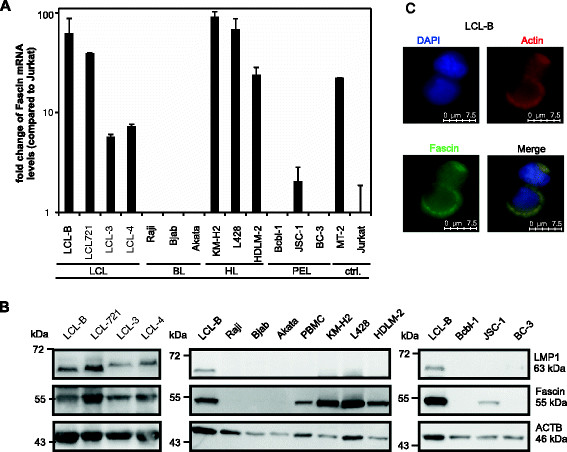
**Expression of Fascin in B-cell lymphomas. (A)** Quantitative PCR (qPCR) of *Fascin* transcripts in transformed B-cells derived from EBV-transformed lymphoblastoid cell lines (LCL), from Burkitt lymphoma (BL), Hodgkin lymphoma (HL), and from primary effusion lymphoma (PEL) in comparison to Jurkat cells and Fascin-positive, HTLV-1-transformed MT-2 cells. Copy numbers were normalized to those of *ß-Actin* (*ACTB*) and thereafter normalized to relative *Fascin* expression in Jurkat cells. The means of two independent experiments are shown. ctrl. indicates control. **(B)** Detection of Fascin and LMP1 by immunoblot. In addition to the B-cell lines shown in **(A)**, peripheral blood mononuclear cells (PBMC) from a healthy donor were analyzed. Detection of ACTB served as loading control. Slower migrating bands upon detection of LMP1 reflect HA-LMP1. **(C)** Immunofluorescence of EBV-transformed LCL-B cells spotted on fibronectin-coated coverslips using phalloidinX-TexasRed for detection of actin and anti-Fascin and secondary anti-mouse Alexa Fluor® 488 antibodies. Nuclei were stained with DAPI. Images were acquired using a LAS AF DMI 6000 fluorescence microscope equipped with a 63 × 1.4 HCX PL APO oil immersion objective lens. Jurkat cells (mock) as shown in Figure 2B served as negative control.

In search of the functional role of Fascin in EBV-transformed lymphocytes, we started to analyze the expression pattern of Fascin in a number of cell lines by quantitative PCR (qPCR; Figure 1A). Human T-lymphotropic virus type 1 (HTLV-1)-transformed MT-2 cells, which express high amounts of *Fascin* [[30]], served as a positive control. In contrast to Jurkat T-cells, which only expressed very low amounts of *Fascin* mRNA, EBV-transformed lymphoblastoid cell lines (LCLs) LCL-B and LCL-721 cells (latency type III) expressed high amounts of *Fascin;* in LCL-3 and LCL-4 (latency type III), expression of *Fascin* was enhanced as well, albeit to lower levels than in LCL-B and LCL 721 cells. Cell lines derived from Hodgkin lymphoma (HL; EBV-negative), including KM-H2, L428, and HDLM-2, expressed high amounts of *Fascin*. All cell lines derived from Burkitt lymphoma (BL; latency type I) did not express *Fascin* confirming earlier observations [[23]]. In B-cell lymphoma cell lines derived from Kaposi’s sarcoma herpes virus-associated malignancies like primary effusion lymphoma (PEL) including EBV-negative cell lines Bcbl-1 and BC-3, and EBV-positive JSC-1 cells (latency type II), *Fascin* was only detectable at low amounts in the PEL-cell line JSC-1. This cell line is known to express low amounts of LMP1, which can be detected by PCR, but not at the protein level [[32]]. Data obtained by qPCR (Figure 1A) were confirmed in immunoblots detecting Fascin protein (Figure 1B). Among all cell lines analyzed, LCL-B, LCL-721, LCL-3 and LCL-4 cells are also LMP1-positive (Figure 1B) [[33]]. Taken together, these results show that expression of Fascin is a specific feature of HL-derived cells [[24]], of LCLs, and of other LMP-1-expressing cell lines (JSC-1). To analyze the subcellular localization of Fascin in transformed, LMP-1 expressing B-cells, immunofluorescence analysis was performed in LCL-B cells (Figure 1C). Fascin was found in the cytoplasm and at the plasma membrane and colocalized with actin, suggesting that Fascin exerts its molecular function of stabilizing actin in EBV-transformed B-cells.

### LMP1 is sufficient to induce Fascin in lymphocytes

**Figure 2:**
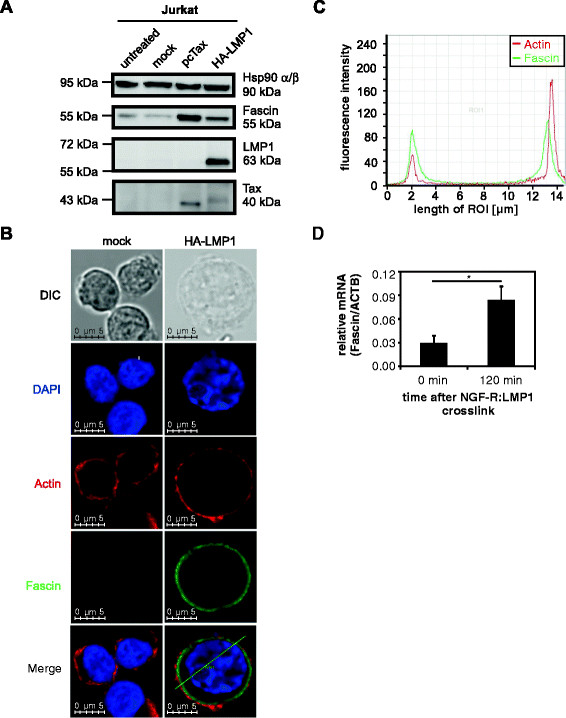
**LMP1 induces Fascin in lymphocytes. (A)** Detection of Fascin by immunoblot of Jurkat cells transiently transfected with wildtype (wt-) LMP1 expression plasmid (pCMV-HA-LMP1) in comparison to mock-transfected (pcDNA3) and Tax-transfected Jurkat cells. As loading control served detection of Hsp90 α/β. **(B)** Triple immunofluorescence staining and confocal microscopy of Jurkat cells transiently transfected with wt-LMP1 expression plasmid (pCMV-HA-LMP1) in comparison to mock-transfected Jurkat cells. Cells were spotted on fibronectin-coated coverslips using phalloidinX-TexasRed for detection of actin and anti-Fascin and secondary anti-mouse Alexa Flour® 488 antibodies. Nuclei were stained with DAPI. Images were acquired using a Leica TCS SP5 confocal laser scanning microscope equipped with a 63 × 1.4 HCX PL APO CS oil immersion objective lens (Leica). DIC indicates differential interference contrast, ROI, region of interest. **(C)** Profiles of the fluorescence intensities of Fascin and actin plotted against the length of the ROI in Jurkat cells expressing LMP1 as shown in **(B)**. **(D)** qPCR of *Fascin* in NGF-R:LMP1 LCLs (B2264-19/3) at the indicated times after cross-linking with anti-NGF-R and anti-fc IgG/IgM antibodies. Copy numbers were normalized to those of *ACTB*. The means of five independent experiments +/−SE were compared (t-test). *indicates P < 0.05.

LMP1 is a potent oncoprotein that contributes to cell transformation and tumor formation by various means (see Introduction). To evaluate whether LMP1 might also be involved in Fascin upregulation observed in LMP1-positive cells, we tested the potential of LMP1 to induce Fascin expression by transfecting Jurkat cells with expression constructs of LMP1 (HA-LMP1) or a mock control (Figure 2A). Jurkat cells were chosen as they express only low levels of endogenous Fascin and they can be transfected efficiently. As a positive control for Fascin induction served Jurkat cells transfected with an expression plasmid for the HTLV-1 Tax oncoprotein, which we previously identified as a specific and strong inducer of Fascin [[30]]. Immunoblot analysis revealed LMP1-mediated Fascin induction. Therefore, not only the HTLV-1-encoded Tax, but also the EBV-encoded LMP1 oncoprotein are potent inducers of Fascin. Immunofluorescence analysis revealed that Fascin localized to the cytoplasm of LMP-1-transfected Jurkat cells (Figure 2B), while mock-transfected cells did not show Fascin expression. Co-staining of actin using TexasRed-coupled phalloidin revealed that Fascin and actin colocalized in LMP1-transfected Jurkat cells, which was further supported by the profiles of the fluorescence intensity for Fascin and actin staining (Figure 2C). These data show that Fascin colocalizes with actin upon LMP1-expression suggesting that both proteins could cooperate in exerting their biological functions. Taken together, the actin-bundling protein Fascin is specifically and strongly upregulated in the presence of EBV/LMP1.

To confirm that Fascin is in fact an immediate-early cellular target gene regulated by LMP1 in EBV-transformed B lymphocytes, the LCL B2264-19/3 expressing a fusion protein of the extracellular and transmembrane domains of the human low-affinity nerve growth factor receptor (NGF-R) and the cytoplasmic signaling domain of LMP1 (NGF-R:LMP1) in the context of the intact EBV genome was analyzed (Figure 2D). B2264-19/3 cells were generated by infection of primary human B-cells with recombinant EBV, in which the wildtype LMP1 gene had been replaced by NGF-R:LMP1 [[34]]. Aggregation of NGF-R:LMP1 at the cell surface by antibodies induces LMP1-specific signaling including activation of NF-κB, p38MAPK, JNK1/2 and STAT1 [[34]]. To induce LMP1 signaling, B2264-19/3 cells were either left untreated or cross-linked with primary antibodies directed against NGF-R and secondary anti-mouse antibodies. After isolation of RNA and cDNA synthesis, qPCR analysis was performed. In contrast to the unstimulated control cells, we observed a significant increase of *Fascin* after 120 min of cross-linking (Figure 2D; p < 0.05; t-test). Monitoring IκBα degradation after NGF-R:LMP1 cross-linking confirmed robust activation of the canonical NF-κB pathway by NGF-R:LMP1 in B2264-19/3 cells (data not shown). Thus, *Fascin* is also a cellular target gene of LMP1 signaling in EBV-infected B-cells.

### CTAR2 of LMP1 is the major site of Fascin induction

**Figure 3:**
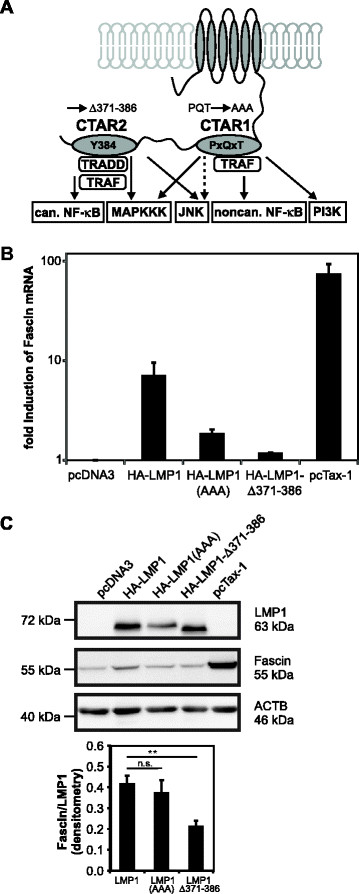
**CTAR2 is the major site of LMP1-mediated Fascin induction. (A)** Scheme of LMP1 and its signaling domains, the C-terminal activating regions 1 (CTAR1; aa 194–231) and 2 (CTAR2; aa 351–386). Mutations of the P(204)xQxT consensus motif to AxAxA in CTAR1 and deletion of aa 371–386 in CTAR2 are indicated. Signaling pathways induced by LMP1 are shown in rectangular boxes, adaptor molecules in rounded boxes. can. indicates canonical; noncan., noncanonical. **(B)** qPCR of *Fascin* transcripts 48 h after transfection of Jurkat cells with wt-LMP1 (20 μg pCMV-HA-LMP1), LMP1 mutants (40 μg pCMV-HA-LMP1(AAA), 20 μg of pCMV-HA-LMP1-Δ371-386) and HTLV-1/Tax (40 μg pcTax-1) in comparison to mock-transfected cells (100 μg pcDNA3). Total transfected DNA was adjusted to 100 μg with pcDNA3. Copy numbers were normalized to those of *ACTB* and on mock-transfected cells. Mean values +/− standard error (SE) are given. **(C)** Detection of Fascin and LMP1 by immunoblot after transient transfection of Jurkat cells with the constructs described in **(B)**. Fascin expression was quantitatively evaluated by densitometry and normalized on LMP1 protein expression. The mean of three independent experiments +/−SE is shown. **indicates *P <* 0.01. n.s., not significant.

LMP1 specifically induces via its cytoplasmatic signaling domains CTAR1 and CTAR2 defined signaling pathways including NF-κB, JNK, PI3K/Akt and p38/MAPKK (Figure 3A). To map the regions in the LMP1 protein that mediate induction of Fascin expression, Jurkat cells were transfected with wt-LMP1, the CTAR1-mutant HA-LMP1(AAA), and the CTAR2-mutant HA-LMP1-Δ371-386. Jurkat cells transfected with pcTax served as a positive control for Fascin induction. Due to differences in LMP1 protein expression levels (data not shown), plasmids encoding LMP1 and the respective mutants had been titrated to reach comparable amounts of LMP1 protein expression. After 48 h, RNA was extracted and upon cDNA synthesis, qPCR was performed (Figure 3B). Compared to mock-transfected cells, LMP1 and Tax-1 induced *Fascin* expression. Whereas expression of HA-LMP1(AAA) led to slight induction of *Fascin*, expression of HA-LMP1-Δ371-386 did not increase *Fascin* mRNA compared to mock-transfected cells, indicating that (1) CTAR2 is essential for LMP1-mediated *Fascin* induction, and (2) CTAR1 contributes to *Fascin* mRNA induction (Figure 3B). To account for different protein expression levels of the LMP1 constructs, protein lysates were isolated in parallel and subjected to Western blot analysis (Figure 3C). Taking into consideration the lower protein expression of HA-LMP1(AAA) compared to wt-LMP1 and the CTAR2-mutant HA-LMP1-Δ371-386, we found that only the CTAR2-mutant was insufficient to induce Fascin protein. Experiments using a CTAR1/CTAR2-double mutant failed as the expression of the double mutant was always much lower than expression of the single mutants alone (data not shown). Therefore, these data show that CTAR1 could contribute to LMP1-mediated *Fascin* induction (Figure 3B), whereas CTAR2 is the major and essential site of Fascin induction (Figure 3B,C).

### LMP1 stimulates Fascin expression via the NF-κB signaling pathway

**Figure 4:**
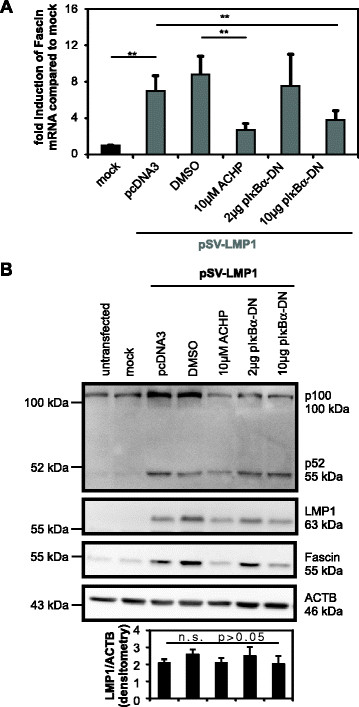
**NF-κB signals are required for LMP1-mediated Fascin induction. (A)** Quantitative PCR of *Fascin* mRNA in Jurkat cells after transfection of wt-LMP1 (pSV40-LMP1) and co-transfection of pIκBα-DN or treatment with the IKKβ inhibitor ACHP (2-Amino-6-(2-(cyclopropylmethoxy)-6-hydroxyphenyl)-4-(4-piperidinyl)-3-pyridine-carboni-trile) solved in DMSO. ACHP (10 μM) was added 24 h after transfection for 24 h. Relative copy numbers were determined by normalizing *Fascin* transcripts to those of *ACTB*. Mean values +/− SE were compared using a t-test (n = 4). **indicates *P <* 0.01. **(B)** Immunoblot of Fascin, LMP1, p100, p52, and ACTB (ß-Actin) in Jurkat cells after transfections as described in (A). LMP1 expression was quantitatively evaluated by densitometry and normalized on ACTB protein expression. The mean of four independent experiments +/−SE is shown and was compared (t-test, n = 4, *P >* 0.05). n.s. indicates not significant.

In addition to activation of the p38 MAPK and JNK pathway [[12],[19]], CTAR2 is required for LMP1-mediated activation of the canonical NF-κB pathway [[8],[15],[16]]. To test whether canonical NF-κB signals are required for LMP1-mediated Fascin induction, LMP1 was either cotransfected with a dominant negative inhibitor of canonical NF-κB signaling, pIκBα-DN (S32/36A) [[35]], or Jurkat cells transfected with LMP1 were incubated in the presence of the IKKβ-inhibitor ACHP. Concentrations of ACHP were chosen such that they are not toxic to Jurkat cells and that they block canonical NF-κB signaling [[36]]. RNA was extracted, subjected to cDNA synthesis and analyzed in qPCR. The presence of pIκBα-DN reduced LMP1-mediated *Fascin* induction dose-dependently (Figure 4A). Upon expression of 10 μg of the IκBα-DN plasmid, LMP1-mediated *Fascin* induction was repressed significantly (p < 0.01; t-test). Block of IKKβ using ACHP (10 μM) also blocked LMP1-mediated *Fascin* induction (p < 0.01; t-test) indicating that NF-κB signals are required for expression of *Fascin*. Quantitation of transcripts of the costimulatory tumor necrosis factor superfamily receptor *4-1BB* (*TNFRSF 9*) in the same samples served as a positive control (Additional file 1). 4-1BB is a target of LMP1 and is induced by CTAR2 requiring canonical NF-κB signals [[37]]. Upon expression of LMP1, *4-1BB* transcripts were induced (p < 0.01, t-test) even at higher magnitudes than *Fascin*. Both ACHP and IκBα-DN (10 μg) resulted in significant reduction of LMP1-mediated *4-1BB* induction, demonstrating the successful repression of canonical NF-κB signals. To further show that canonical NF-κB signals are required for LMP1-mediated Fascin induction, protein expression of LMP1 and Fascin was analyzed in western blot analysis (Figure 4B). Detection of p100 and its processing into p52 served as controls for the activity of canonical and non-canonical NF-κB signaling, respectively (Figure 4B). LMP1 led to an increase in p100 expression and p52 processing, reflecting activity of both NF-κB signaling pathways. However, in the presence of ACHP and IκBα-DN, only p100 was reduced, while processing of p100 into p52 was unaffected, indicating that canonical NF-κB signals were selectively blocked. In consistency with the data observed on *Fascin* transcript levels (Figure 4A), also Fascin protein (Figure 4B) was reduced by coexpression of pIκBα-DN (10 μg). Moreover, inhibition of IKKβ by ACHP also abrogated LMP1-mediated induction of Fascin protein. Despite a slight but insignificant influence of inhibitor treatment on LMP1 protein expression as measured by densitometry (Figure 4B; n = 4; n.s., p > 0.05), *Fascin* was reduced significantly in the presence of NF-κB inhibitors (Figure 4A). Taken together, in addition to a functional CTAR2 domain (Figure 3), an intact canonical NF-κB signaling pathway is required for induction of Fascin by LMP1 in transfected cells.

**Figure 5:**
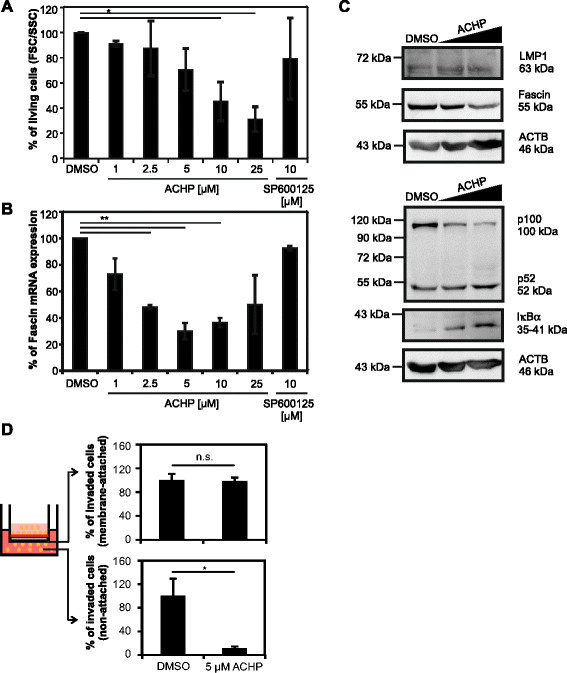
**NF-κB signals are required for maintaining Fascin expression and invasive migration of EBV-transformed LMP1-expressing lymphoblastoid cells (LCL). (A)** Viability of LCL-B cells upon treatment with increasing amounts of the IKKβ inhibitor ACHP (1, 2.5, 5, 10, 25 μM) and the JNK-inhibitor SP600125 (10 μM) for 48 h determined by forward-side scatter (FSC/SSC) analysis in flow cytometry. DMSO-treated cells were set as 100%. The means of three independent experiments +/− SE were compared using a paired t-test. *indicates *P <* 0.05. **(B)** Quantitative PCR of *Fascin* transcripts normalized to *ACTB* in LCL-B upon ACHP-and SP600125-treatment for 48 h. The means of three independent experiments +/− SE were normalized to solvent-treated cells and compared using a paired t-test. **indicates *P <* 0.01. **(C)** Detection of Fascin, LMP1, p100 processing (p100/p52) and IκBα by immunoblot after treatment of LCL-B with DMSO, 2.5 and 5 μM ACHP for 48 h. ACTB served as loading control. Samples were loaded on two gels in parallel. **(D)** LCL-B cells were cultured in presence of ACHP (5 μM) or solvent (DMSO) for 48 h and cells were serum-starved (1% fetal calf serum (FCS)) for 4 h. Invasion assays were performed using trans-wells coated with extracellular matrix for 24 h. Values shown in the upper panel reflect the percentage of invaded cells (measured at OD 560 nm) that are attached to the bottom of the membrane. The lower bar graphs show the percentage of invaded cells that are non-attached and have migrated to the medium (20% FCS) of the lower compartment. Solvent-treated cells (DMSO) were set as 100%. Mean values and error bars of three independent experiments each performed in triplicates are shown. Values were compared using a paired t-test. *indicates P < 0.05. n.s., not significant.

### The NF-κB signaling pathway is required for Fascin expression and invasive migration of EBV-transformed, LMP1-expressing lymphoblastoid cells

To analyse whether canonical NF-κB signals are also required for Fascin expression in EBV transformed LMP1-expressing B-cells, LCL-B cells were incubated with increasing amounts of the IKKβ-inhibitor ACHP (Figure 5A-C). Treatment of cells with a selective inhibitor of the JNK pathway (SP600125; 10 μM) served as specificity control [[38]]. After 48 h, viability of cells was determined by flow cytometry and RNA was extracted. Forward-side-scatter (FSC/SSC) analysis revealed that low concentrations of ACHP (1, 2.5, 5 μM) only slightly affected viability of the LCL-B culture compared to the solvent control DMSO (p > 0.05; paired t-test; Figure 5A). However, high concentrations of ACHP (10, 25 μM) reduced viability of LCL by 50-75% (p < 0.05; paired t-test) confirming earlier observations [[39]]. Quantitation of *Fascin* copy numbers by qPCR showed that even at low concentrations of ACHP (2.5, 5 μM), *Fascin* copy numbers were significantly and dose-dependently reduced (Figure 5B; p < 0.01; paired t-test), while inhibition of JNK signaling with SP600125 did not affect *Fascin* expression. To ensure specificity of the IKKβ-inhibitor ACHP in LCLs, transcripts of the NF-κB-dependent LMP1-target gene *4-1BB* were measured (Additional file 2) [[37]]. Already at low concentrations of ACHP (1μM), expression of *4-1BB* was diminished significantly (p < 0.01). While *Fascin* was only affected by treatment with ACHP, *4-1BB* was also diminished upon treatment with the JNK-inhibitor SP600125, which confirms earlier findings showing a role of both NF-κB and JNK signaling in 4-1BB regulation [[40]]. To further address the influence of NF-κB signals on Fascin protein, Western blot analysis was performed upon treatment of LCLs with low doses of ACHP (2.5 μM; 5 μM). These data revealed that also Fascin protein is reduced upon treatment of LCLs with ACHP, despite the presence of LMP1 (Figure 5C). Beyond that, treatment of LCLs with ACHP led to less production of p100, a classical target of canonical NF-κB signaling, while processing of p100 to p52 was not affected. Finally, we observed an accumulation of IκBα, suggesting that (1) IκBα gets less degraded in presence of ACHP, and that (2) canonical NF-κB signals are blocked. In summary, these data show that Fascin is regulated by canonical NF-κB signals not only in LMP1-transfected cells, but also in LMP1-expressing, EBV-transformed lymphoblastoid B-cells.

Fascin contributes to invasion of cancer cells [[28],[29],[41]] and HTLV-1-transformed T lymphocytes [[30]], however, the relative contribution of Fascin to the motility of EBV-transformed lymphocytes has not been investigated. To analyse whether inhibition of NF-κB, which leads to reduction of Fascin (Figure 5C), also affects invasion of EBV-transformed lymphocytes, LCL-B cells were incubated in the presence of ACHP (5 μM, 48 h) and serum-starved for 4 h. Subsequently, invasion assays were performed utilizing basement-membrane coated inserts which separate the cells from medium with 20% fetal calf serum (FCS) in the lower well. Invasive cells are able to degrade the matrix, pass through the pores of the polycarbonate membrane, and attach either to the bottom of the membrane (Figure 5D, upper panel), or they migrate to the lower well after invasion (Figure 5D, lower panel). We did not detect different numbers of cells attached to the bottom of the membrane (Figure 5D, upper panel; n = 3; t-test; p > 0.05). This suggests that inhibition of NF-κB does not affect adhesion of invaded LCLs to the membranes used in our assay. However, we observed that the number of invaded and non-attached LCLs in the lower well was significantly reduced to approximately 11% in presence of ACHP compared to the solvent control (Figure 5D, lower panel; n = 3; t-test, p < 0.05). We observed slight reduction of cell vitality in presence of the inhibitor (Figure 5A, compare 5 μM ACHP and DMSO; p > 0.05), but we measured significant impairment of NF-κB activity and Fascin expression (Figure 5B, C). Therefore, we conclude that inhibition of NF-κB significantly reduces the migratory rate of LCLs subsequent to invasion of the extracellular matrix, and Fascin might contribute to this phenotype.

### Knockdown of Fascin reduces the invasive capacity of LMP1-expressing lymphocytes.

**Figure 6:**
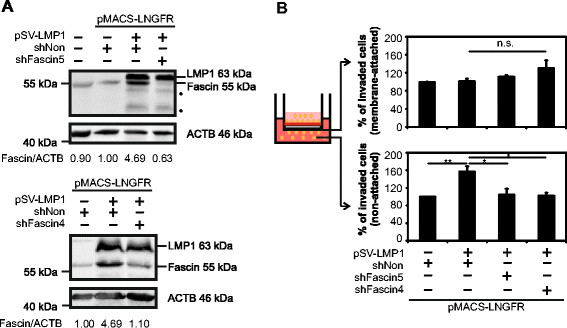
**Knockdown of Fascin in LMP1-transfected Jurkat cells diminishes invasion through extracellular matrix. (A)** Jurkat cells were transfected with pMACS-LNGFR, wt-LMP1 (pSV-LMP1) and shFascin5, shFascin4 or shNonsense (shNon) and subjected to magnetic separation. Immunoblots of Fascin, LMP1 and ACTB are shown. Numbers indicate the relative expression of Fascin normalized on ACTB as determined by densitometry (at least 4 experiments). Values obtained from cells transfected with LMP1 and a control shRNA (shNon) were set as 1. indicates cross reactions of the LMP1-antibody. **(B)** Invasion assays were performed with serum-starved Jurkat cells (1% FCS) after magnetic separation using trans-wells coated with extracellular matrix for 24 h. Values shown in the upper panel reflect the percentage of invaded cells (measured at OD 560 nm) that are attached to the bottom of the membrane. The lower bar graphs show the percentage of invaded cells that are non-attached and have migrated to the medium (20% FCS) of the lower compartment. Mock-transfected cells were set as 100%. Mean values and error bars of three independent experiments each performed in quadruplicates are shown. Values were compared using a paired t-test. **indicates P < 0.01. *indicates P < 0.05. n.s., not significant.

In studies focusing on NPC and cells of epithelial origin, LMP1 has been described as a potent regulator of cellular migration and invasion [[42]–[45]]. To test, whether sole expression of LMP1 induces invasion of lymphocytes, too, and whether this specifically depends on Fascin, invasion assays were performed in transiently transfected cells. For this purpose, Jurkat cells were transfected with LMP1-expression plasmids, two different shRNA-constructs targeting Fascin (shFascin4; shFascin5) or unspecific control shRNAs (shNonsense). To increase the sensitivity of our analysis, cells were co-transfected with an expression plasmid for LNGFR, which encodes a cytoplasmic truncated, low-affinity nerve growth factor receptor that is not expressed on Jurkat cells (data not shown), and therefore allows positive selection of transfected cells by magnetic separation. Flow cytometry using LNGFR-specific antibodies revealed that the amount of LNGFR-expressing cells was enriched by magnetic separation from approximately 33% to ca. 82%, independent of the combination of co-transfected plasmids (Additional file 3). Upon enrichment, a robust Fascin induction by LMP1 was observed in the presence of non-targeting control shRNA (Figure 6A, 4.69-fold), whereas co-expression of shFascin5 or shFascin4 caused a knockdown of Fascin with an efficiency of 87% (from 4.69 to 0.63) or 77% (from 4.69 to 1.10), respectively. Cells were serum-starved for 5 h in 1% FCS and invasion assays were performed utilizing basement-membrane coated inserts which separate the cells from medium with 20% FCS in the lower well as described in Figure 5D. Although we did not detect a significantly increased number of cells attached to the bottom of the membrane (Figure 6B, upper panel; n = 3; t-test; p > 0.05), we observed that expression of LMP1 significantly enhanced the number of invaded and non-attached Jurkat cells in the lower well to approximately 158% compared to the mock control (100%); (Figure 6B, lower panel; n = 3; t-test, p < 0.01). Functional knockdown of Fascin using shFascin 5 or shFascin 4 reduced the amount of invaded, non-attached cells to 105% or 103%, respectively (n = 3; t-test, p < 0.05), demonstrating that Fascin strongly contributes to the increasing number of cells migrated to the lower well. Therefore, our data suggest that neither LMP1 nor Fascin affect adhesion of invaded lymphocytes to the membranes used in our assay. However, LMP1 enhances the migratory rate of Jurkat cells subsequent to invasion of the extracellular matrix, and Fascin accounts primarily for this phenotype. Taken together, we conclude that the viral oncoprotein LMP1 is sufficient to induce the tumor marker Fascin dependent on canonical NF-κB signals, which could contribute to invasive migration.

## Discussion

The tumor marker Fascin is an actin-bundling protein related to migration and invasion in an increasing number of neoplastic diseases [[28],[29],[41]]. Here we show that the EBV-encoded oncogene LMP1 induces the tumor marker Fascin in lymphocytes. Induction of Fascin by LMP1 strongly depends on an intact CTAR2 domain as demonstrated by ectopic expression of LMP1-mutants. Canonical NF-κB signaling plays an important role in LMP1-mediated induction of Fascin in both transfected and transformed, LMP1-expressing lymphocytes. In functional analyses, we show that canonical NF-κB signaling and Fascin expression contribute to invasive migration of LMP1-expressing lymphocytes through the extracellular matrix.

There has been evidence that Fascin is expressed in EBV-transformed lymphoblastoid cell lines (LCLs) [[23]], which is confirmed in this study. Our data showing that *Fascin* is a cellular target gene immediately induced by LMP1 signaling in LCLs could explain this phenotype. In contrast, EBV-positive Burkitt Lymphoma (BL)-derived cell lines, which are known to be LMP1-negative [[1],[2]], do not express Fascin. A different situation exists for Hodgkin’s lymphoma (HL)-derived cells used in our study, which express high amounts of Fascin although they are LMP1-negative. Expression of Fascin had been described earlier in cutaneous CD30^+^ lymphoproliferative disorders [[46]], and in HL-derived Reed-Sternberg cells [[24]]. Fascin was discussed as a prognostic marker of HL [[24]]. Despite the absence of LMP1, both the canonical and noncanonical NF-κB pathways are constitutively activated in HL due to genetic lesions, auto-and paracrine signals, and expression of TNF receptor (TNFR) family members [[47]]. Moreover, aberrant activation of the NF-κB pathway is of key importance for the survival of HL-derived cells [[47]]. Therefore, constitutive activation of NF-κB could explain high expression levels of Fascin in the absence of LMP1 in HL-derived cells requiring further investigation. On the other hand, NF-κB activity does not automatically result in expression of Fascin as both Bjab and primary effusion lymphoma (PEL) cells do not express Fascin despite high levels of NF-κB activity [[48],[49]]. However, our data show that NF-κB is necessary for Fascin induction by LMP1 and Fascin expression in LMP1-transformed LCLs, but it may not be sufficient in other types of transformed B-cells.

Our findings show a direct link between LMP1 expression and the induction of Fascin in both B and T lymphocytes. These observations are in line with findings describing the presence of Fascin in lymph node metastases in NPC. Fascin expression positively correlated with the expression of both LMP1 and the phosphorylated transcription factor signal transducer and activator of transcription 3 (STAT3), as well as with the proliferation index of the tumor cells [[25]]. Collectively, LMP1-mediated induction of Fascin may not only be restricted to lymphocytes but also be applicable to cells of epithelial origin, which suggests that LMP1-mediated induction of Fascin is a general phenomenon of EBV-biology.

LMP1 is not only expressed in latently infected B-cells, but can also be upregulated during the lytic cycle in both epithelial cells and B-cells. LMP1 seems to play a role in virus production, as LMP1-deleted EBV enters the lytic replication cycle as efficiently as the wild-type counterpart, but is severely impaired in virus release into culture supernatants [[50]], pointing to a defect in particle transport. LMP1-mediated expression of the actin-bundling protein Fascin in the cytoskeleton and its continuous expression suggest a role of Fascin in virus release. This is further corroborated by the finding that cell-to-cell transmission of EBV to epithelial cells also depends on canonical NF-κB signaling [[51]], which is also a prerequisite for efficient Fascin induction.

Our data showing enhanced invasive migration of lymphocytes in the presence of Fascin suggest that EBV exploits functions of Fascin. The capacity of Fascin to induce migration of tumor cells could also be relevant to the migratory capacity of EBV-transformed cells and to EBV-associated disease, however, it remains to be determined whether Fascin is essential for invasive migration of LCLs, as it is in LMP1-expressing Jurkat cells. Our data show that block of canonical NF-κB signaling reduces both Fascin and invasive migration of EBV-transformed LCLs, thus, strengthening the assumption that Fascin contributes to invasive migration of LCLs, too. However, canonical NF-κB signaling also affects the expression of other proteins than Fascin that could contribute to cellular motility as well. Yet, selective repression of Fascin in LMP1-expressing Jurkat T lymphocytes revealed that in this cell type Fascin contributes to invasive migration. As yet, it was known that LMP1 is a potent regulator of cellular migration and invasion since LMP1 is capable of inducing a wide range of cellular factors involved in tumor metastasis [[42]]. Both LMP1-mediated transcriptional, posttranscriptional and posttranslational regulation of cellular targets could contribute to the capacity of LMP1 to promote spreading of tumor cells: (1) LMP1 causes loss of junctional plakoglobin in nasopharyngeal carcinoma (NPC) cells and initiates a cadherin switch [[52]]. (2) LMP1 upregulates decoy receptor 3, a member of the TNFR superfamily, which enhances NPC cell migration and invasion [[43]]. (3) LMP1 down-regulates E-cadherin gene expression and induces cell migration activity by using cellular DNA methylation machinery [[45]]. (4) In NPC cells, LMP1 increases phosphorylation of the membrane cross linker ezrin through a protein kinase C (PKC) pathway. Recruitment of ezrin to the cell membrane linked to F-actin and CD44 is a process required for LMP1-stimulated cell motility and invasion of NPC cells [[42]]. We now show that LMP1 can also induce the actin-bundling Fascin, which is strongly associated with migration and invasion in many types of cancer [[27]–[29]]. In contrast to previous studies, which mainly focused on cells of epithelial origin and NPC [[42],[43],[45]], we now show in T lymphoid cells that LMP1 is also important for invasive migration, whereas it seems to be dispensable for attachment of invaded cells. Beyond that our data highlight for the first time an important role of Fascin in LMP1-mediated invasive migration. Interestingly, LMP1’s capacity to enhance migration is regulated by PI3K/Akt and also by IκBα-dependent canonical NF-κB signaling in NPC cells [[44]]. Thus, LMP1-mediated induction of NF-κB also appears to contribute to LMP1-induced cell migration in lymphocytes, in particular by regulation of Fascin.

Activation of the NF-κB pathway is linked to LMP1-induced immortalization of primary B lymphocytes. Although signaling via CTAR2 mainly induces canonical NF-κB signaling and production of p100, CTAR2 is not sufficient for transformation in the absence of CTAR1. In contrast, CTAR1 is only a weak activator of NF-κB and induces noncanonical NF-κB signaling resulting in processing of p100, but is sufficient for initial transformation (reviewed in [[6]]). We show by three approaches that canonical NF-κB signals are important for LMP1-mediated Fascin induction: (1) A mutation of CTAR2 that is defective in NF-κB-signaling [[33]] failed to induce Fascin, (2) Use of a super-repressor of NF-κB blocked LMP1-mediated Fascin-induction, and (3) chemical block of IKKβ reduced canonical NF-κB signaling and Fascin expression in both LMP1-transfected and LMP1-transformed lymphocytes. Earlier studies have shown that Fascin harbours κB consensus sites in its promoter [[53]], and we have shown that Fascin expression can be induced by NF-κB [[30]]. Contribution of NF-κB to expression of Fascin was also confirmed in a breast cancer cell line showing binding of p65 to the Fascin promotor [[54]]. Collectively, these findings suggest that LMP1 regulates Fascin expression via canonical NF-κB signaling not only in lymphocytes, but potentially also in other cell types.

We have previously shown that Fascin expression can be induced by the viral oncoprotein Tax of the tumor virus Human T-lymphotropic virus type 1 (HTLV-1), which belongs to the family of delta-retroviridae [[30]]. Beyond that, we found a novel mode of transcriptional regulation of Fascin showing the importance of NF-κB signaling in Tax-mediated Fascin induction [[30]]. Therefore, the LMP1-mediated induction of Fascin via NF-κB signaling may be a common mechanism of lymphotropic tumor viruses revealing a new quality of virus-induced oncogenesis. All tumor viruses with naturally occurring distinct oncogenes reprogram persistently infected cells in the direction of growth promotion and survival functions, and it is plausible that these are side effects of viral growth and propagation. Now, we have shown that not only the leukemia-inducing retrovirus HTLV-1, but also the oncogenic herpesvirus EBV can induce Fascin. However, future studies are needed to address whether other viral oncoproteins like the KSHV-encoded oncoprotein vFLIP, which activates both canonical and non-canonical NF-κB pathways [[49]], are able to induce Fascin. In contrast to LCLs, PEL cells do not express Fascin, suggesting that regulation of Fascin does not only depend on cell type and on the NF-κB signaling pathway, but also on other properties of different viral oncoproteins.

## Conclusions

Here we report for the first time that LMP1 induces Fascin in lymphocytes and this depends on canonical NF-κB signaling. Fascin mediates invasiveness of carcinoma cells, a typical function of tumor progression. Our data indicate a contribution of Fascin to invasive migration of LMP1-expressing lymphocytes. Collectively, our findings suggest that Fascin plays a role in viral oncogenesis.

## Methods

### Cell culture

Cell lines used in this report include the Epstein-Barr virus-positive (EBV^+^) human lymphoblastoid B-cell lines (LCLs) LCL-B (provided by G. Niedobitek) and LCL-721 [[55]]; the EBV^+^ LCLs LCL-3 and LCL-4, which are derived from *in vitro* transformation of human B lymphocytes with a recombinant maxi-EBV in which the wildtype *LMP1* gene had been replaced by *HA-LMP1* [[33]]; the LCL clone B2264-19/3 expressing chimeric nerve growth factor receptor (NGF-R):LMP1 allowing inducible LMP1 signaling [[34]]; the EBV-negative (EBV^−^) Hodgkin lymphoma (HL)-derived cell lines KM-H2 [[56],[57]], L428 [[58]], and HDLM-2 [[59],[60]]; the EBV^+^, Burkitt Lymphoma (BL)-derived B-cell line Raji [[61]]; the EBV^−^, BL-derived B-cell line Bjab [[62]], and the EBV^−^ B-cell line Akata (an Akata subclone that has lost the virus) [[63]]; the EBV^−^, Kaposi’s sarcoma-associated herpesvirus-positive (KSHV^+^) B-cell lines Bcbl-1 [[64]] and BC-3 [[65]] derived from primary effusion lymphoma (PEL); the EBV^+^ KSHV^+^ PEL-derived B-cell line JSC-1 [[32]]; the Human T lymphotropic virus type 1 (HTLV-1) *in vitro* transformed CD4^+^ T-cell line MT-2 [[66]]; and the acute lymphoblastic leukemia T-cell line Jurkat [[67]]. LCL-B-cells were cultured in RPMI 1640M containing fetal calf serum (FCS), 50 μM β-mercaptoethanol (GIBCO, Life Technologies, Darmstadt, Germany), 1 mM sodium pyruvate (GIBCO), glutamine, and penicillin/streptomycin. LCL-721, LCL-3, LCL-4, and MT-2 cells were cultured in RPMI 1640M, 10% FCS, glutamine (0.35 g/L) and penicillin/streptomycin. B2264-19/3 B-cells expressing NGF-R:LMP1 were cultivated on γ-irradiated CD40L-expressing fibroblast feeder cells in RPMI 1640M containing 10% FCS, 100 nM sodium selenite (Sigma-Aldrich), 1% sodium pyruvate, 0.5 mM monothioglycerol (Sigma-Aldrich), 0.02 μM bathocuproinedisulfonic acid (Sigma-Aldrich) and penicillin/streptomycin. All other cell lines were cultured in RPMI 1640M containing 45% Panserin 401 (PAN-Biotech, Aidenbach, Germany), 10% FCS, glutamine (0.35 g/L) and gentamycine. Peripheral blood mononuclear cells (PBMC) were isolated from buffy coats of anonymized healthy donors (Institut für Transfusionsmedizin, Suhl, Germany) by Ficoll-Hypaque gradient centrifugation (Biocoll, Biochrom, Berlin, Germany). Informed consent was not requested as the data were analyzed anonymously and the samples had not been collected specifically for this study. This procedure was approved by the Ethics Committee of the Medical Faculty of Friedrich-Alexander-Universität Erlangen-Nürnberg (Erlangen, Germany). PBMC were cultured in RPMI 1640M containing 10% FCS, glutamine, penicillin/streptomycin, phytohemagglutinin (PHA-P; 2 μg/ml; Sigma-Aldrich) and interleukin-2 (25 U/ml) for 48 h.

### Construction of shRNA expression vectors

For knock-down of Fascin by RNA interference (RNAi), the retroviral shRNA expression vectors pSiren-IRES-EGFP-shFascin5 (shFascin5) [[30]], and pSiren-IRES-EGFP-shFascin4 (shFascin4) were constructed. Oligonucleotides for shRNAs were designed with the *siRNA Hairpin Oligonucleotide Sequence Designer Tool* (Clontech). They contained (5′ to 3′) a *Bam*HI site, the respective siRNA sequence (bold), a loop region, the complementary siRNA sequence (bold), an RNA polymerase III termination sequence, an *Mlu*I restriction enzyme site (italicized), and an *Eco*RI cloning site (shFascin4-fwd: 5′-gatccG**CAAAGACTCCACAGGCAAA**TTCAAGAGA**TTTGCCTGTGGAGTCTTTG**TTTTTT*ACGCGT*g-3′; shFascin4-rev: 5′-aattc*ACGCGT*AAAAAA**CAAAGACTCCACAGGCAAA**TCTCTTGAA**TTTGCCTGTGGAGTCTTTGC**g-3′) Oligonucleotides were annealed in 10 mM Tris and 20 mM NaCl (pH 7.6) by heating to 95°C for 2 min followed by cooling to room temperature. Double-stranded oligonucleotides were thereafter inserted into the retroviral vector pSiren-IRES-EGFP-shNonsense (shNon) [[68]] using T4 ligase (DNA *Ligation kit*, TaKaRa Biomedicals, Gennevilliers, France) after removal of the shNon fragment via *Bam*HI and *Eco*RI restriction sites. The resulting shRNA expression plasmid was called pSiren-IRES-EGFP-shFascin4 (target at position +1407 of the Fascin coding sequence, gene bank accession number NM_003088).

### Immunoblots

Protein lysates were obtained by lysis of cells in 150 mM NaCl, 10 mM Tris pH 7.0, 10 mM EDTA, 1% Triton, 2 mM dithiothreitol (DTT) and protease inhibitors (20 μg/ml Leupeptin, 20 μg/ml aprotinin and 1 mM phenyl-methyl-sulfonyl fluoride). After repeated *freeze-and-thaw* cycles, equal amounts of protein were denatured for 5 min at 95°C in sodium dodecyl sulfate (SDS) loading dye (10 mM Tris pH 6.8, 10% glycerine (w/v), 2% SDS (w/v), 0.1% bromphenol blue (v/v), 5% β-mercaptoethanol (v/v)) and subjected to SDS- polyacrylamide gel electrophoresis (SDS-PAGE) followed by immunoblotting on *Nitrocellulose Transfer Membranes* (Whatmann ®, PROTRAN ®, Whatmann GmbH, Dassel, Germany). Immunoblots were probed using the rabbit monoclonal antibody anti-NF-κB2 p100/p52 (18D10; 1:1000, Cell Signaling Technology, MA, USA) and mouse monoclonal antibodies anti-Fascin (55K-2; 1:1000; Dako Deutschland GmbH, Hamburg, Germany), anti-β-actin (ACTB; AC-15; 1:2500; Sigma, Taufkirchen, Germany), anti-Hsp90α/β (F-8; 1:1000; Santa Cruz Biotechnology, Heidelberg, Germany), anti-LMP1 (clones CS.1-4; 1:100; Dako, Hamburg, Germany), anti-IκB-α (H-4; 1:1000; Santa Cruz Biotechnology) and mouse antibodies to Tax (1:50), which were derived from the hybridoma cell line 168B17-46-34 (provided by B. Langton through the AIDS Research and Reference Reagent Program, Division of AIDS, NIAID, NIH) [[69]]. Secondary antibodies conjugated with horseradish peroxidase were obtained from GE Healthcare (Little Chalfont, UK). Peroxidase activity was detected by enhanced chemiluminescence using a *Kodak Image Station 4000MM PRO camera* (Kodak). In some experiments, proteins were blotted on PVDF membranes (Immobilon-FL, Merck Millipore, Billerica, MA, USA) pre-incubated in methanol and goat anti-mouse Alexa Fluor® 647-labelled secondary antibodies (1:2000; Life Technologies) were used. Fluorescence intensity was detected using *Kodak Image Station 4000MM PRO camera*. At least three independent experiments were performed and one representative result is shown. Intensities of specific bands were quantitated using *Advanced Image Data Analyser* (AIDA Version 4.22.034, Raytest Isotopenmessgeräte GmbH, Straubenhardt, Germany) and the mean of at least three independent experiments is shown.

### Immunofluorescence and confocal laser scanning microscopy

Cells were spotted on 10 μg/mL fibronectin-coated (Sigma) coverslips, fixed with 4% para-formaldehyde (20 min), washed twice with PBS and permeabilized with 0.2% Triton X-100 (20 min, 4°C). After four wash steps, unspecific binding was blocked by 5% FCS/ 1% BSA in PBS (1 h, 20°C). Cells were incubated with anti-Fascin mouse monoclonal antibodies (1:100; Dako) for 30 min at 37°C. After washing, cells were incubated with Alexa Fluor® 488-conjugated goat anti-mouse IgG secondary antibodies (Life Technologies) for 30 min at 37°C. For double-labelling with filamentous actin, cells were co-incubated with Texas Red-X phalloidin (1:20; Life Technologies). For staining of nuclei, cells were incubated with VECTASHIELD Mounting Medium with DAPI (Vector Laboratories, Burlingame, CA, USA). Images were acquired using a LAS AF DMI 6000 fluorescence microscope equipped with a 63 × 1.4 HCX PL APO oil immersion objective lens (Leica Microsysteme Vertrieb GmbH, Wetzlar, Germany). Alternatively, images were acquired using a Leica TCS SP5 confocal laser scanning microscope equipped with a 63 × 1.4 HCX PL APO CS oil immersion objective lens (Leica). Images were analyzed and signal intensities were quantified using LAS AF software (Leica).

### Quantitative real-time RT-PCR (qPCR)

Total cellular RNA was isolated from cell lines or transfected cells (*RNA isolation Kit II*, Macherey-Nagel, Düren, Germany; *RNeasy micro Kit*; Qiagen, Hilden, Germany) and reversely transcribed to cDNA using *Superscript II* and random hexamer primers (both Life Technologies GmbH, Darmstadt, Germany) or *QuantiTect Reverse Transcription Kit* (Qiagen, Hilden, Germany). Quantitative real-time RT-PCR (qPCR) was performed in an *ABI Prism 7500 Sequence Analyzer* (Applied Biosystems, Foster City, CA) using 200 ng of cDNA and *SensiMix*^*™*^*II Probe Kit* (Bioline GmbH, Luckenwalde, Germany) according to the manufacturer’s instructions. Primers and FAM (6-carboxyfluorescein)/TAMRA (tetramethylrhodamine)-labeled probes for detection of *β-actin* (*ACTB*) transcripts and *4-1BB* have been described before [[70]]. For quantitation of *Fascin* transcripts, a *TaqMan Gene Expression Assay* (Hs00979631_g1; Applied Biosystems) was used. Expression levels were computed by interpolation from standard curves generated from plasmids carrying the respective target sequences and calculating the mean of triplicate samples. Each sample was measured in at least three biological replicates. *ACTB* was used for normalization.

### Inhibitor treatment of LCL-B

LMP1-positive, EBV-transformed LCL-B cells were incubated with increasing amounts (0, 2.5 μM, 5 μM, 10 μM; 25 μM) of an inhibitor of IκB kinase β (IKK-β), ACHP (2-Amino-6-(2-(cyclopropylmethoxy)-6-hydroxyphenyl)-4-(4-piperidinyl)-3-pyridinecarboni-trile); Calbiochem/ Merck, Darmstadt, Germany), dissolved in DMSO. After 48 h, RNA was extracted and viability of cells was determined analyzing forward versus side scatter (FSC vs. SSC) using an *BD Accuri C6* flow cytometer (BD Biosciences, San Jose, CA, USA). A JNK-specific inhibitor SP600125 (10 μM) was used as control. Protein lysates were obtained from cells after treatment with DMSO and ACHP (2.5 μM, 5 μM) for 48 h.

### Transient transfection by electroporation

10^7^ Jurkat T-cells were transfected by electroporation using *Gene Pulser X® Electroporation System* (BioRad, Munich, Germany) at 290 V and 1500 μF with 20 μg pCMV-HA-LMP1, 40 μg pCMV-HA-LMP1(AAA), 20 μg pCMV-HA-LMP1-Δ371-386 [[16],[33]] or 40 μg pcTax-1 [[30]]. pCMV-HA-LMP1(AAA) is mutated in CTAR1 and the PxQxT TRAF-binding motif is substituted by alanines (AxAxA) [[16]], while HA-LMP1-Δ371-386 carries a deletion of the carboxy-terminal cytoplasmic region in CTAR2 and is incapable of recruiting TRADD and TNIK [[18],[33]]. Total transfected DNA was adjusted to 100 μg with pcDNA3 (Life Technologies GmbH). In experiments where NF-κB signaling was blocked, 10^7^ Jurkat cells were transfected with 40 μg of an SV40-promoter-driven LMP1-construct, pSV-LMP1 [[19]], and 2 μg or 10 μg of a dominant negative inhibitor of IκBα (pIκBα-DN; S32/36A), a plasmid carrying two mutations at critical serine residues S32 and S34 that are usually phosphorylated by IKKβ, thereby leading to proteasomal degradation of IκBα [[35]]. Total transfected DNA was adjusted to 50 μg with pcDNA3. In transient transfections, the IKK-β-inhibitor ACHP (2.5 μM; 10 μM) was added 24 h post transfection for 24 h. Cells were harvested 48 h after transfection to isolate RNA and to perform immunoblots. For invasion assays, Jurkat cells were transfected with 10 μg pMACS-LNGFR (Miltenyi Biotec, Bergisch Gladbach, Germany), 40 μg pSV-LMP1 [[19]], 20 μg pSiren-RetroQ-IRES-EGFP-shNonsense (sh Nons), pSiren-RetroQ-IRES-EGFP-shFascin5 (shFascin 5), or pSiren-RetroQ-IRES-EGFP-shFascin4 (shFascin 4). Total transfected DNA was adjusted to 100 μg with pcDNA3.

### Cross-linking of NGF-R:LMP1

Prior to cross-linking of NGF-R:LMP1, B2264-19/3 cells were cultivated in the absence of CD40L feeder cells for three days. For NGF-R-cross-linking the cells were incubated in culture medium supplemented with 1 μg/ml anti-NGF-R for 30 minutes at 37°C. Cross-linking was performed in the presence of 10 μg/ml anti-fc IgG/IgM (115-005-068; Dianova) for the indicated times as described [[34]].

### Magnetic separation

To enrich LMP1-expressing cells, Jurkat cells co-transfected with pMACS-LNGFR were washed with PBS (without Ca^2+^ and Mg^2+^) 48 h post transfection, and stained with anti-LNGFR-PE conjugated antibodies (ME20.4-1.H4; 1:10; Miltenyi Biotec) for 10 min (4°C), followed by an incubation with anti-PE *MicroBeads* (Miltenyi Biotec) for 15 min (4°C). Labeled cells were separated using *MACS LS columns* (Miltenyi Biotec) on a *MidiMACS™* Separator (Miltenyi Biotec). The percentage of cells stained for LNGFR was determined with the *BD Accuri C6* flow cytometer (BD Biosciences) before and after magnetic separation.

### Invasion assay

After magnetic separation LNGFR-enriched Jurkat cells were serum-starved in cell culture medium containing 1% FCS for 4 h. LCL-B cells were cultured in presence of 5 μM ACHP or DMSO for 48h prior to serum starvation. Invasion assays were performed using *CytoSelect™24-Well Cell Invasion Assay* (colorimetric format, Cell Biolabs Inc., San Diego, CA) according to the manufacturer’s instructions. Briefly, cells were counted and 2 × 10^5^ Jurkat cells or 1.5 × 10^5^ LCL-B cells in 300 μl medium (1% FCS) were applied to the upper chamber of a trans-well containing polycarbonate membranes with 8 μm pore sizes covered with extracellular matrix isolated from mouse Engelbreth-Holm-Swarm sarcoma. The lower chamber contained cell culture medium supplemented with 20% FCS. Cells were incubated at 37°C for 24 h. After aspirating media from the inside of the insert and cleaning the inside with cotton-tipped swabs, the inserts were stained with *Cell Stain Solution*, washed and extracted with *Extraction Solution*. Finally the OD 560 nm of the cell extraction solution was measured with *E*_*max*_*precision microplate reader* (MWG-Biotech GmbH, Ebersberg, Germany) reflecting the amount of invaded cells attached to the bottom of the membranes. At least three independent experiments were performed in quadruplicates (Jurkat) or triplicates (LCL-B). Invaded cells in the lower compartment (non-attached cells) were counted in at least four visual fields using a Neubauer chamber in quadruplicates (Jurkat) or triplicates (LCL-B) in at least three independent experiments.

### Statistics

SPSS version 16.0.2 (SPSS, Chicago, IL) was used for statistical analysis using the t-test. *P <* 0.05 was considered to be significant.

## Abbreviations

ACHP: 2-Amino-6-(2-(cyclopropylmethoxy)-6-hydroxyphenyl)-4-(4-piperidinyl)-3-pyridine-carbonitrile

ACTB: β-actin

BL: Burkitt lymphoma

CTAR: C-terminal activation region

DTT: Dithiothreitol

EBV: Epstein-Barr virus

FAM: 6-carboxyfluorescein

FCS: fetal calf serum

FSC: Forward scatter

FSCN1: Fascin

HHV-4: Human herpesvirus 4

HL: Hodgkin lymphoma

HTLV-1: Human T-lymphotropic virus type 1

IKK: IκB kinase

LCL: Lymphoblastoid cell line

JNK: c-Jun N-terminal kinase

LMP1: Latent membrane protein 1

LNGFR: Low-affinity nerve growth factor receptor

NGF-R: Nerve growth factor receptor

NIK: NF-κB inducing kinase

NF-κB: Nuclear factor kappa B

NPC: Nasopharyngeal carcinoma

PEL: Primary effusion lymphoma

SDS: Sodium dodecyl sulfate

SSC: Side scatter

STAT: Signal transducer and activator of transcription

TAMRA: Tetramethylrhodamine

TNFR: Tumor necrosis factor receptor

TRADD: TNF-receptor associated death domain

TRAF: Tumor necrosis factor receptor-associated factor

## Competing interests

The authors declare that they have no competing interests.

## Authors’ contributions

CFM and MK contributed equally to this study and performed most of the experiments. CG, MCM, and KRS performed experiments. AK and BF provided essential contributions to the study. CFM, AK and BF participated in writing the manuscript. AKK designed and supervised the study and wrote the manuscript. All authors read and approved the final manuscript.

## Additional files

## Supplementary Material

Additional file 1: **NF-κB signals are required for LMP1-mediated induction of *4-1BB.*** Quantitative PCR of *4-1BB* mRNA in Jurkat cells after transfection of wt-LMP1 (pSV40-LMP1) and co-transfection of pIκBα-DN or treatment with the IKKβ inhibitor ACHP (2-Amino-6-(2-(cyclopropylmethoxy)-6-hydroxyphenyl)-4-(4-piperidinyl)-3-pyridine-carboni-trile) solved in DMSO. ACHP (10 μM) was added 24 h after transfection for 24 h. Relative copy numbers were determined by normalizing *4-1BB* transcripts to those of *ACTB*. Mean values +/− SE were compared using a t-test (n = 4). * indicates *P <* 0.05; **, *P <* 0.01. Click here for file

Additional file 2: **NF-κB signals are required for maintaining expression of *4-1BB* in lymphoblastoid cells.** Quantitative PCR of *4-1BB* transcripts normalized to *ACTB* in LCL-B upon ACHP-and SP600125-treatment for 48 h. The means of three independent experiments +/− SE were normalized to solvent-treated cells and compared using a paired t-test. ** indicates *P <* 0.01. Click here for file

Additional file 3: **Enirchment of transfected cells by magnetic separation.** FACS analysis of transfected Jurkat cells before and after magnetic separation. Jurkat cells were transfected with pMACS-LNGFR, wt-LMP1 (pSV-LMP1) and shFascin5, shFascin4 or shNonsense (shNon). Cells were stained for LNGFR expression and subjected to magnetic separation. The percentage of LNGFR-positive cells (mean values +/− SE) is shown (at least 4 experiments). Click here for file
