# Supplementary figures and images for: The tumor marker Fascin is induced by the Epstein-Barr virus-encoded oncoprotein LMP1 via NF-κB in lymphocytes and contributes to their invasive migration
Source: Cell Commun Signal. 2014 Jul 11;12:46. doi: 10.1186/s12964-014-0046-x (PMC4222691; doi:10.1186/s12964-014-0046-x)

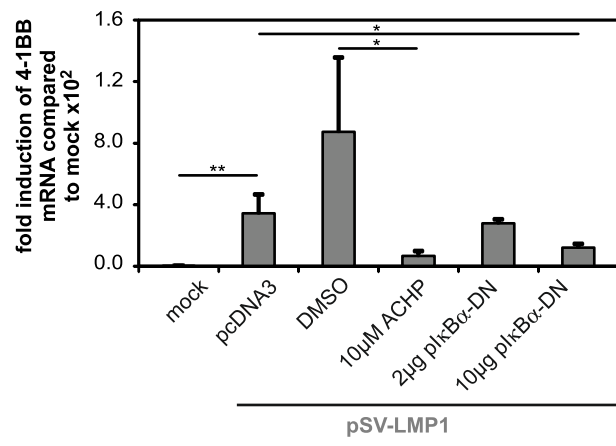

Supplement: Additional file 1: — NF-κB signals are required for LMP1-mediated induction of 4-1BB. Quantitative PCR of 4-1BB mRNA in Jurkat cells after transfection of wt-LMP1 (pSV40-LMP1) and co-transfection of pIκBα-DN or treatment with the IKKβ inhibitor ACHP (2-Amino-6-(2-(cyclopropylmethoxy)-6-hydroxyphenyl)-4-(4-piperidinyl)-3-pyridine-carboni-trile) solved in DMSO. ACHP (10 μM) was added 24 h after transfection for 24 h. Relative copy numbers were determined by normalizing 4-1BB transcripts to those of ACTB. Mean values +/− SE were compared using a t-test (n = 4). * indicates P < 0.05; **, P < 0.01. [file s12964-014-0046-x-S1.pdf]

**A**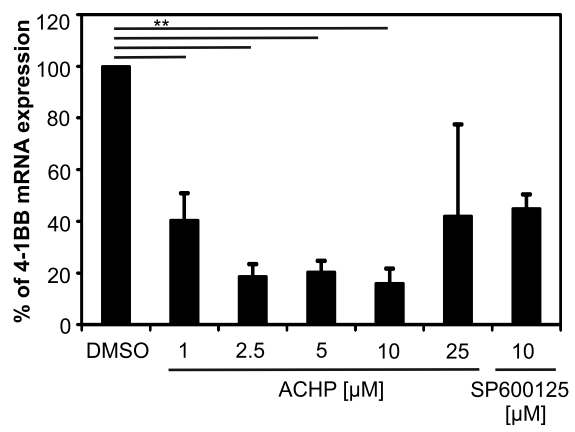

Supplement: Additional file 2: — NF-κB signals are required for maintaining expression of 4-1BB in lymphoblastoid cells. Quantitative PCR of 4-1BB transcripts normalized to ACTB in LCL-B upon ACHP-and SP600125-treatment for 48 h. The means of three independent experiments +/− SE were normalized to solvent-treated cells and compared using a paired t-test. ** indicates P < 0.01. [file s12964-014-0046-x-S2.pdf]

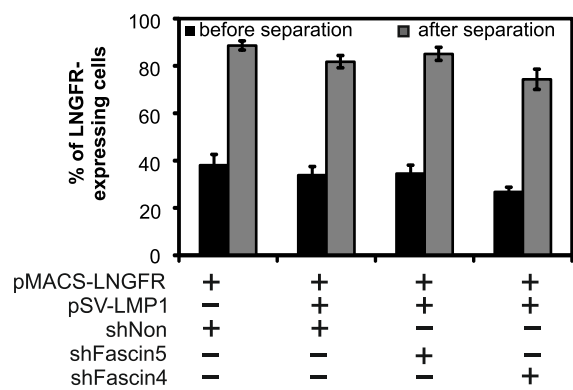

Supplement: Additional file 3: — Enirchment of transfected cells by magnetic separation. FACS analysis of transfected Jurkat cells before and after magnetic separation. Jurkat cells were transfected with pMACS-LNGFR, wt-LMP1 (pSV-LMP1) and shFascin5, shFascin4 or shNonsense (shNon). Cells were stained for LNGFR expression and subjected to magnetic separation. The percentage of LNGFR-positive cells (mean values +/− SE) is shown (at least 4 experiments). [file s12964-014-0046-x-S3.pdf]
